# AI Literacy in Health‐Professions Education: Deepening the Case for Curricular Reform in the Philippines

**DOI:** 10.1002/hsr2.71551

**Published:** 2025-11-18

**Authors:** Aaron A. Funa, Renz Alvin E. Gabay

**Affiliations:** ^1^ College of Health and Science Sorsogon State University Sorsogon City Sorsogon Philippines; ^2^ College of Teacher Education Sorsogon State University Sorsogon City Sorsogon Philippines

Dear Editor,

In response to Mousavi Baigi et al. [1] in *Health Science Reports*, we argue that Philippine health‐professions programs should move from ad hoc exposure to structured, competency‐based AI education. While the authors document broadly positive student attitudes, they also reveal conceptual gaps and uneven preparedness; these findings have two immediate implications for curriculum design and assessment. First, attitude homogeneity across subgroups (e.g., medical vs. paramedical) does not imply equivalent competence; shared exposure to general‐purpose tools can mask differences in foundational knowledge, evaluation skills, and ethical judgment. Second, self‐reported familiarity can easily be confounded with what the authors call “conceptual limitations,” which—if left unaddressed—become patient‐safety risks once AI tools are embedded in clinical workflows. These nuances strengthen, not weaken, the authors' call for structured AI education that moves beyond ad hoc exposure to principled instruction and supervised practice.

The Philippine higher‐education landscape mirrors these tensions. Analyses from the University of the Philippines Center for Integrative and Development Studies describe an uneven institutional response: campuses have begun issuing guidance on responsible, transparent AI use, but sector‐wide uptake remains patchy pending Commission on Higher Education (CHED) actions and program‐level redesign [2]. Critically, the recommendations go beyond “tool training.” They argue for embedding AI literacy as an explicit learning outcome, integrating discipline‐specific applications, and redesigning assessment to make documented AI use legitimate and auditable—precisely the levers needed to convert student optimism into durable competence.

Healthcare raises the stakes. Recent guidance for Philippine healthcare leaders highlights structural constraints (connectivity, data stewardship, workforce capacity) and governance requirements (bias and safety monitoring, transparency, accountability) that must accompany any deployment [3]. For educators, this translates into curricular tasks that are not optional add‐ons but core clinical competencies: interpreting model outputs, identifying bias and drift, documenting human–AI decision pathways, and practicing escalation when AI recommendations conflict with clinical judgment. The same guidance calls for national strategy and capacity‐building aligned to international standards—context that should anchor program‐level outcomes and accreditation expectations for medicine, nursing, and allied health.

Two strands of Philippine scholarship help operationalize these goals. A meta‐synthesis of AI‐in‐education policies consolidates actionable themes—AI literacy as a measurable outcome, ethics and academic integrity embedded across courses, assessment redesign for transparent AI use, and sustained institutional support through faculty development and access [4]. These are not generic slogans; they map onto teachable, assessable competencies in health‐professions education (e.g., critique of model performance, data governance plans in capstone projects, and viva‐style defenses of AI‐assisted clinical reasoning). In parallel, a cross‐generational study of Philippine science educators shows heterogeneous familiarity and confidence across cohorts, implying that faculty development must be tiered (baseline, intermediate, advanced) and mentored rather than one‐size‐fits‐all—vital for colleges where senior clinicians supervise AI‐enabled learning [5].

Building on these sources (and without changing the core findings of Mousavi Baigi et al.), we propose a three‐layer framework tailored to the Philippine context:

a. Layer 1—Foundations (preclinical or first‐year): Define AI types, data lifecycles, and limitations; practice reading model cards and basic performance metrics; introduce bias, fairness, and privacy with Filipino cases (e.g., class imbalance in local registries). Short, supervised labs should require students to articulate when not to use AI and how to document AI assistance in clinical notes. This directly addresses the “conceptual limitations” flagged in the target study [1].
b. Layer 2—Applied judgment (clinical years): Integrate AI into case conferences using structured “human‐on‐the‐loop” checklists; require error analysis (false‐positive/false‐negative impact on Filipino patients) and mitigation strategies; and implement assessment rubrics that reward transparent, ethical AI use rather than undetectable outsourcing. These align with policy recommendations on assessment redesign and integrity [4].
c. Layer 3—Governance and systems (internship/residency bridge): Teach data governance (access controls, provenance), risk management (model drift, post‐deployment monitoring), and accountability (who signs off when humans and models disagree). Capstone projects can partner with hospitals to pilot safe, auditable workflows, aligning graduate capabilities with national healthcare‐AI recommendations [3].

Implementation details matter. Philippine programs should: (a) publish program‐level AI outcomes mapped to CHED outcomes and accreditation standards; (b) scaffold academic‐integrity practices specific to AI (declarations, logs of prompts and outputs, and reflective justifications); and (c) invest in staged faculty development that recognizes generational differences while building a community of practice across medicine, nursing, and allied health. These measures are consistent with both higher‐education reform proposals and sector‐specific governance advice [2, 3, 4].

In sum, the contribution of Mousavi Baigi et al. is a springboard—evidence that enthusiasm is real but unevenly matched by understanding. Filipino educators can respond by embedding foundational literacy, applied judgment, and governance skills across the health‐professions curriculum, ensuring that graduates are not merely AI‐ready but patient‐ready in an AI‐enabled health system.

## Author Contributions

Both authors drafted the letter and approved the final version.

## Ethics Statement

This letter involves no human participants or patient data.

## Conflicts of Interest

The authors declare no conflicts of interest.

## Transparency Statement

This letter is an honest and accurate commentary on the cited work.

## Data Availability

The authors have nothing to report.
